# Cohort Profile: The Shaanxi Blood Donor Cohort in China

**DOI:** 10.3389/fcvm.2022.841253

**Published:** 2022-05-11

**Authors:** Lei Zhang, Hengxin Li, Shu Su, Erica M. Wood, Ting Ma, Yang Sun, Lingxia Guo, Qianke Cheng, Xiaoyun Gu, Wenjie Wu, Liqin Wang, Miao Ding, Leilei Zhang, Yuan Shen, Jiangcun Yang

**Affiliations:** ^1^Department of Transfusion Medicine, Shaanxi Provincial People’s Hospital, Xi’an, China; ^2^China-Australia Joint Research Center for Infectious Diseases, School of Public Health, Xi’an Jiaotong University Health Science Center, Xi’an, China; ^3^Melbourne Sexual Health Centre, Alfred Health, Melbourne, VIC, Australia; ^4^Department of Epidemiology and Biostatistics, College of Public Health, Zhengzhou University, Zhengzhou, China; ^5^Blood Quality Management Office, Shaanxi Provincial Blood Center, Xi’an, China; ^6^Transfusion Research Unit, School of Public Health and Preventive Medicine, Monash University, Melbourne, VIC, Australia; ^7^Data Center, Shaanxi Provincial People’s Hospital, Xi’an, China; ^8^Planning Development and Information Office, Health Commission of Shaanxi Province, Xi’an, China; ^9^Department of Information Technological, Shaanxi Health Information Center, Xi’an, China; ^10^Department of Epidemiology and Biostatistics, School of Public Health, Xi’an Jiaotong University Health Science Center, Xi’an, China

**Keywords:** cohort profile, blood donation, ICD-10, health effects, China

## Abstract

**Purpose:**

The Shaanxi Blood Donor Cohort was set up to investigate the impact of blood donation on the health of donors compared with non-blood donors. The specific aims of the study include (1) identifying the geographical and temporal trends of incidence for diseases in both blood donors and non-blood donors; (2) assessing the impact of environmental exposures, lifestyle, body mass index (BMI) and blood type on disease burdens, stratified between blood donors and non-blood donors; and (3) among blood donors, investigating if regular blood donation has a positive impact on donors’ health profiles, based on a cohort with a mixed retrospective and prospective study design.

**Participants:**

A total of 3.4 million adults, with an equal number and identical demographic characteristics (year of birth, sex and location of residence) of blood donors and non-blood donors, were enrolled on 2012. The one-to-one matching was conducted through a repeated random selection of individuals without any history of blood donation from the Shaanxi Electronic Health Records. The cohort has been so far followed up to the end of 2018, summing to nearly 24 million years of follow-up. The cohort will be followed up prospectively every 3 years until 2030.

**Findings to Date:**

Of the 1.7 million blood donors, 418,312 (24.5%) and 332,569 (19.5%) individuals were outpatients and inpatients, accounting for 1,640,483(96.2%) outpatient and 496,061 (29.1%) inpatient visits. Of the same number of non-blood donors, 407,798 (23.9%) and 346,097 (20.3%) individuals were hospital outpatients and inpatients, accounting for 1,655,725 (97.1%) outpatient and 562,337 (33.0%) inpatient visits. The number of outpatient and inpatient visits by non-blood donors was 0.9 and 3.9% higher than those of the blood donors (*p* < 0.01). Blood donors demonstrate significantly fewer inpatients visits than non-blood donors for major chronic disease categories (*p* < 0.01).

**Future Plans:**

We are currently exploring the long term benefits of blood donation on major chronic disease categories and multimorbidities in this large population cohort. The study results are adjusted by the “healthy donor effect.” This cohort study will continue until 2030.

## Introduction

Blood donation saves lives and is essential for clinical care (1). A “healthy donor effect” undoubtedly exists, in that donors are overall healthier, at the time of donation, than non-donors (2). This is likely, in part, due to pre-donation health screening requirements but may also reflect other factors, including socioeconomic status. However, whether blood donation also provides health benefits to donors as a consequence of donation is less clear. For example, reducing iron overload through regular blood donation may prevent cancer, hemochromatosis, and liver diseases (3, 4), but iron depletion and iron deficiency with anemia are common among frequent blood donors (5–7). To date, there is insufficient scientific evidence to identify the impacts of blood donation on donors’ health at a population level. We, therefore, established a large natural population cohort consisting of an equal number of both blood donors and non-blood donors of comparable demographics to assess the impacts of blood donation on donors’ subsequent disease burden following blood donation.

Previous investigations have not provided conclusive evidence on the health impact of donation on blood donors. While some studies have demonstrated that regular blood donation may reduce the occurrence of cardiovascular and cerebrovascular events (8–10), others reported that the cardiovascular benefits of blood donation (for example, by depleting lipid-oxidizing iron) are controversial (11, 12). Cohort studies among disqualified donors could not demonstrate any reduction in the incidence of coronary disease or myocardial infarction (13, 14). One observational study with 292 subjects found regular blood donation is associated with decreases in blood pressure in individuals with hypertension (10), but a separate study indicated no differences (15). Blood donation has not shown protective effects for cancer development, but findings were confounded by many factors such as smoking, alcohol consumption, body mass index, physical activity, and occupation exposures (4, 16, 17). Most of these studies were longitudinal studies or cohort studies with a relatively short follow-up period and small sample size, which is insufficient to distinguish the differences in disease burden between blood donors and non-blood donors. As most clinical symptoms after blood donation were self-reported, recall bias also undermined the results of these studies (18, 19).

To our best knowledge, only a few cohort studies investigate the association between blood donation and its subsequent impact on disease burden in a population (20–22). We address this knowledge gap by establishing a combined retrospective and prospective cohort in a large Chinese population. The cohort was recruited in the Chinese province of Shaanxi, with a permanent resident population of 39 million in 2019. We collected information on 3.4 million blood donors from the Shaanxi Blood Donor Database during 1998–2018. By linking it with the province-wide Shaanxi Electronic Health Records (EHR) and Centralized Hospital Medical Records (CHMRs), provides an unprecedented opportunity to investigate the potential benefits/adversities of blood donation for any clinically diagnosed diseases (defined by ICD-10) in donors (compared with non-donors) in a natural and representative population cohort.

The key objectives of this cohort study include (1) identifying the geographical and temporal trends of incidence for both communicable and non-communicable diseases in both blood donors and non-blood donors; (2) assessing the impact of environmental exposures, lifestyle, body mass index (BMI) and blood type on disease burdens, stratified between blood donors and non-blood donors; and (3) among blood donors, investigating if regular blood donation has a positive impact on donors’ health profiles.

## Methods and Analysis

### Cohort Participants

**FIGURE 1:**
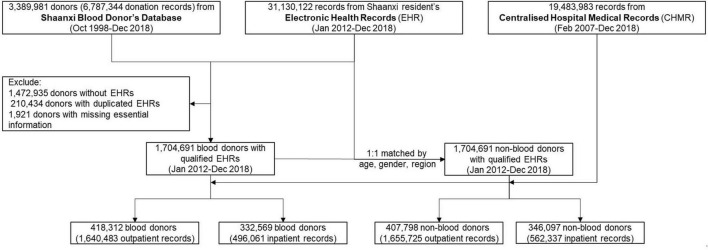
Flowchart demonstrates the steps of cohort construction. The inclusion criteria of cohort participants, (1) blood donors are recorded in both Shaanxi Blood Donor’s Database and Shaanxi resident’s Electronic Health Records; (2) blood donors have a unique identification number to enable them to be linked across datasets; (3) each non-blood donor is matched to a blood donor with identical age, gender and location of residence.

**FIGURE 2:**
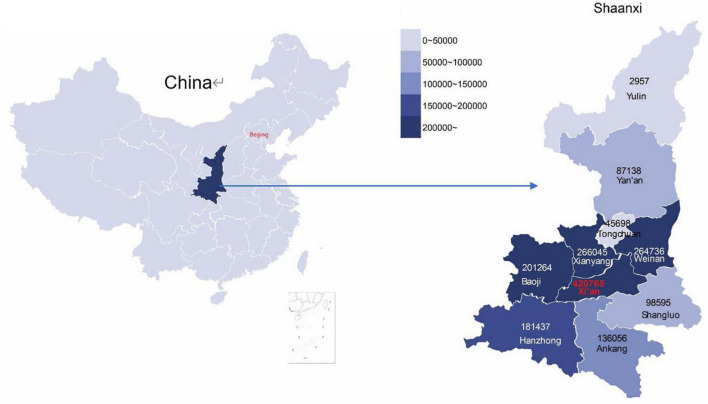
The geographical distribution of enrolled blood donors in the cohort in Shaanxi province, China. The enrolled non-blood donors share an identical distribution.

We constructed this cohort through comprehensive data linkages of major population health databases in Shaanxi province. The Shaanxi Blood Donor’s Database was established in 1998 and recorded all voluntary blood donation events in the province. All blood stations on the prefecture-level adopted a standardized blood collection and information management system. During 1998–2018, a total of 67,367,713 donation event records from 3,389,981 blood donors were collected (Figure 1). The database covered all ten prefectures in Shaanxi (Figure 2). The key variables collected at the point of blood donation included information related to demographics, the process of blood donation, anthropometric measures, and medical indicators (Table 1). The Whole Blood and Component Donor Selection requirements by China’s Ministry of Health excluded individuals from blood donation if they have one of the following medical conditions: chronic respiratory diseases, circulatory system diseases, urinary system diseases, autoimmune diseases, blood system diseases, chronic skin disease, allergic diseases, neurological, and mental diseases, infectious diseases, parasite disease, cancers and certain occupational diseases (23). Individuals who previously received a transplant were not eligible for donation. The requirements classified 16 health conditions for temporary deferrals, including dental cleaning, tooth extraction, surgery, abortion, delivery, breastfeeding, upper respiratory infection, acute gastroenteritis, receipt of blood components or products, and recent vaccination (24). Donors are required to have a minimum weight of 50 kg for males and 45 kg for females and a normal systolic (90–140 mm Hg) and diastolic (60–90 mmHg) blood pressure. The donation interval requirement is at least 6 months apart between whole blood donations and 2 weeks apart for apheresis donation, and the legal age for donation was 18–55 years (23). Hemoglobin and blood counts were measured prior to the donation according to the Chinese guidelines for blood donation (25).

**TABLE 1:** Summary of data collected in the Shaanxi Blood Donor Cohort, based on the linkage of three major health datasets.

Shaanxi blood donor’s database	Electronic health records	Centralized Hospital Medical Records
**Pre-donation**	Hemoglobin count	Blood count
**Blood donation data**	**Demographic data**	**Inpatient records**
Blood type	Birthdate	Date of hospital admission
Blood type (retested)	Sex	Counts of hospital admission (ever)
Time since last blood donation	Ethnicity	Hospitalization history
Number whole blood donation (ever)	Marital status	Date of hospital discharge
Number of all-type blood donation (ever)	Registered address	Department of discharge
Volume of blood donation	Current residential address	Discharge status (recovered or death)
Volume of the last blood donation	**Socioeconomic data**	Diagnosed diseases (primary, ICD-10)
Year of blood donation	Education	Diagnosed complications (up to 9, ICD-10)
Type of blood donation (apheresis or whole blood donation)	Occupation	Medical transfer records
**Anthropometric survey, blood pressure and pulse**	Health insurance (Y/N)	Medical transfer from community health services/rural health
Height (cm)	**Health behaviors**	Total hospitalization costs
Weight (kg)	Smoking status	Surgical operation
BMI	Drinking status	**Outpatient records**
Waist (cm)	Physical activity	Date of outpatient visits
Blood pressure (mmHg)	Dietary habits	Hospital attended
Pulse rate (beats/minute)	**Personal medical history**	Diagnosed diseases (primary, ICD-10)
Laboratory testing of donated blood	Disease diagnosis records	Diagnosed complications (up to 4, ICD-10)
Rh (D)	Diagnosis date	Outpatient visit expenses
ALT	**Family history**	
HBsAg	Parental medical history of diabetes	
HCV	Parental medical history of hypertension	
TP	Parental medical history of coronary heart disease	
HIV RNA	Parental medical history of stroke	
HBV DNA	Parental medical history of psychosis	
HCV DNA	Parental medical history of tumor	
NAAT for sexually transmitted infections	**Operation, injury and blood transfusion data**	
	Type of operation, injury and blood transfusion	
	Date of operation, injury and blood transfusion	
	**Death records**	
	Date of death	
	Cause of death	

The Shaanxi Blood Donor Database was subsequently integrated with Shaanxi residents’ Electronic Health Records (EHR), administered by Shaanxi Provincial Health Planning Policy Evaluation and Information Center. EHR was a key digital health initiative implemented nationwide by the Chinese government in 2009 and initiated in Shaanxi in 2012 (26). In 2012, approximately 50% of the 40 million Shaanxi’s residents were covered by EHR. Its objective was to maintain continuous and trackable medical records for its residents and provide health stakeholders with evidence for disease trends and distribution. EHR collected basic health information from locally registered residents and residents from other Chinese provinces who have lived in Shaanxi for more than 6 months. As of 2018, 70% of the Shaanxi residents have registered in EHR (27). The rollout of EHR was determined by the geographical differences in willingness and overall planning of implementation. The records included basic demographic information, past personal and family medical history, health examinations, and key population health management records (Table 1). EHR data was collected through household surveys, disease screening, health check-ups, and records from community health service centers. The EHR data was used as the baseline information for the cohort and subsequently linked to the hospital notification data. Our inclusion criteria are to recruit all the blood donors with an EHR record in the cohort. We, thus, matched the Shaanxi Blood Donor Database with the EHR based on unique resident identification numbers. The linkage resulted in 1,917,046 EHR records matched to the blood donor dataset (Figure 1), and have excluded 1,472,935 donors without EHR. Further, after excluding 210,434 duplications and 1,921 entries that miss essential information, 1,704,691 blood donors were found in files of EHRs and included in this study.

Of the available 31,130,122 records in EHR, we created a non-blood donor cohort with individuals of an identical year of birth, sex, and residency location as the blood donor cohort. The one-to-one matching was conducted through a repeated random selection of individuals without any history of blood donation from the EHR and comparing the three characteristics with those from the blood donor cohort. During the process, the same non-blood donor cannot be selected more than once. The selection process was repeated until all 1,704,691 non-blood donors were selected (Figure 1). The selection of identical years of birth and sex removed the potential confounding effects caused by these demographic characteristics on disease burden. In contrast, the identical residency location selection ensures that the geographical distributions of the diseases between the two cohorts are comparable (Table 1).

The combined cohort of blood donors and non-blood donors was further linked to the Centralized Hospital Medical Record (CHMR) of Shaanxi Province, also administered by Shaanxi Provincial Health Planning Policy Evaluation and Information Center. In China, hospital outpatient consultations and inpatient admission were compulsorily reportable for state-funded health insurance schemes (28). As of 2018, CHMR covered 473 public hospitals, 702 private hospitals and 625 community hospitals in Shaanxi. In CHMR, the outpatient records included the date of visit, primary diagnosis and associated complications, and consultation and examination expenses. For inpatients, medical records also include counts of hospital admissions, history of hospitalization, discharge status (discharged or death), department of discharge, and discharge dates or transfer information. All outpatients and inpatients records include patients’ residential identification numbers, which enabled a data linkage with the already integrated blood donor and non-blood donor datasets. As a result, of the 1,704,691 blood donors, a total of 496,061 inpatient records and 1,640,483 outpatient records were identified; of the same number of non-blood donors, 538,803 hospital records and 1,655,725 outpatient records were identified (Figure 1). All records’ identification numbers and identifiable information were removed after linkage by the data custodian and blinded from the researchers.

In summary, we established a retrospective cohort of 1.7 million blood donors and 1.7 million non-blood donors with identical demographic characteristics for the period 2012–2018. We anticipate following up and expanding this cohort prospectively every 3 years during 2019–2030.

### Cohort Follow-Up

The cohort was initiated in 2012 at the same time when both standard EHR and CHMR databases were first established as required by the Chinese government. EHR and CHMR databases were updated yearly when new records were received. Although the first record of CHMR could be dated back to 2007, the information was only uploaded automatically by only very few hospitals and the uploaded data had no standardized format between hospitals. Since 2012, CHMR data has been standardized and initially covered 80% of specialized hospitals and 30% of primary hospitals. This coverage has increased to 100% for specialized hospitals and 80% for primary hospitals in 2018 (Supplementary Table 2). We excluded the CHMR before 2012 to avoid bias in data. The timing of the first record of EHR was regarded as the baseline for cohort participants. The cohort was retrospectively followed until the end of 2018, which accounted for a total of 23,780,396 person-years of follow-up accumulatively. During the follow-up, we regarded the first record of disease in the CHMR database as the onset of the disease unless the participant had self-reported the disease’s pre-existence at baseline. We estimated disease incidence as the ratio of the number of participants who developed the disease and the overall follow-up duration. Similarly, we calculated disease mortality as the ratio of the number of participants who died of the diseases and the overall follow-up duration.

Starting from 2019, the existing cohort of 3.4 million participants will continue to be followed up every 3 years until the end of 2030 (2021, 2024, 2027, and 2030). The cohort remains open in each follow-up, and new blood donors are allowed to enroll into the cohort in 2021, 2024, and 2027. When new blood donors are added to the cohort, an equal number of non-blood donors with identical years of birth, sex, and residence location will be randomly selected from EHR and enrolled in the cohort. The same EHR and CHMR indicators will be updated regularly in each follow-up. The design of the prospective cohort will add to the assessment of the temporal trend of the disease burden, particularly disease incidence, in the cohort.

Cohort participants were considered to reach their follow-up endpoints if they satisfied one of the following: (1) one of EHR and CHMR have indicated that the individual has died; (2) the participant’s EHR record has not been updated in three consecutive years; (3) participant’s current residential address on EHR is no longer within Shaanxi province.

### Findings to Date

The equal number of blood donors and non-blood donors of identical age (in years), sex distribution and geographical distribution (Tables 2A,B) reflect their matching demographic characteristics. The overall cohort is relatively young, with a mean age of 36.4 (*SD* ± 10.6) years and predominantly male (59.0%), and resided in the Central Shaanxi region (71.5%). Both male and female blood donors and non-blood donors demonstrate similar marital status, with married individuals accounting for approximately 60% of the participants, followed by 25–30% who were never married. However, blood donors seem to be better educated (Senior high and above) and have a lower proportion of peasants as their occupations than non-blood donors (χ^2^-test, all *p* < 0.01).

**TABLE 2:** Basic demographic characteristics of blood donors and matched non-blood donors in the Shaanxi Blood Donor Cohort at the time of enrolment.

(a) Male blood donors			
	**Blood donors (%)**	**Non-blood donors (%)**	**χ ^2^-test (*P*-value)**
Number of participants	1,003,082	1,003,082	
**Age, years**	36.7 ± 10.3	36.7 ± 10.3	−
18–24	97,946 (9.8)	97,946 (9.8)	
25–29	185,444 (18.5)	185,444 (18.5)	
30–34	206,034 (20.5)	206,034 (20.5)	
35–39	144,094 (14.4)	144,094 (14.4)	
40–44	118,565 (11.8)	118,565 (11.8)	
45–49	116,882 (11.7)	116,882 (11.7)	
50–54	74,156 (7.4)	74,156 (7.4)	
55–59	40,344 (4.0)	40,344 (4.0)	
60–64	15,071 (1.5)	15,071 (1.5)	
65–69	3,872 (0.4)	3,872 (0.4)	
=70	674 (0.1)	674 (0.1)	
Average donation counts	1.8 ± 0.8	0	
**Education**			< 0.01
Junior high and below	526,493 (52.5)	596,736 (59.5)	
Senior high	330,487 (32.9)	255,361 (25.5)	
University and above	42,373 (4.2)	35,358 (3.5)	
Unknown	103,729 (10.3)	115,627 (11.5)	
**Marriage status**			< 0.01
Single	265,452 (26.5)	306,754 (30.6)	
Married	678,515 (67.6)	611,918 (61.0)	
Divorced	8,519 (0.8)	9,999 (1.0)	
Unknown	50,596 (5.0)	74,411 (7.4)	
**Occupation**			< 0.01
Student	20,035 (2.0)	22,477 (2.2)	
Worker	141,656 (14.1)	85,540 (8.5)	
Peasant	575,083 (57.3)	647,924 (64.6)	
Self-employed	248,194 (24.7)	231,885 (23.1)	
Unknown	18,114 (1.8)	15,256 (1.5)	
**Region**			
North Shaanxi	45,884 (4.6)	45,884 (4.6)	
Central Shaanxi	758,788 (75.6)	758,788 (75.6)	
South Shaanxi	198,410 (19.8)	198,410 (19.8)	

**(b) Female blood donors**			

	**Blood donors (%)**	**Non-blood donors (%)**	**χ ^2^-test (*P*-value)**
Number of participants	701,609	701,609	
**Age, years**	36.0 ± 11.0	36.0 ± 11.0	−
18–24	91,773 (13.1)	91,773 (13.1)	
25–29	142,077 (20.3)	142,077 (20.3)	
30–34	157,838 (22.5)	157,838 (22.5)	
35–39	74,711 (10.6)	74,711 (10.6)	
40–44	64,151 (9.1)	64,151 (9.1)	
45–49	71,144 (10.1)	71,144 (10.1)	
50–54	51,449 (7.3)	51,449 (7.3)	
55–59	31,232 (4.5)	31,232 (4.5)	
60–64	13,366 (1.9)	13,366 (1.9)	
65–69	3,168 (0.5)	3,168 (0.5)	
=70	700 (0.1)	700 (0.1)	
**Average donation counts**	1.6 ± 0.9	0	
**Education**			< 0.01
Junior high and below	313,920(44.7)	357,891(51.0)	
Senior high	232,061 (33.1)	208,315 (29.7)	
University and above	42,862 (6.1)	31,125 (4.4)	
Unknown	112,766 (16.1)	104,278 (14.9)	
**Marriage status**			< 0.01
Single	178,526 (25.4)	186,466 (26.6)	
Married	453,191 (64.6)	569,997 (67.0)	
Divorced	5,119 (0.7)	1,934 (0.3)	
Unknown	64,773 (9.2)	43,212 (6.2)	
**Occupation**			< 0.01
Student	49,857 (7.1)	35,482 (5.1)	
Worker	95,296 (13.6)	101,976 (14.5)	
Peasant	340,336 (48.5)	374,891 (53.4)	
Self-employed	198,435 (28.3)	167,013 (23.8)	
Unknown	17,685 (2.5)	22,247 (3.2)	
**Region**			-
North Shaanxi	58,102 (8.3)	58,102 (8.3)	
Central Shaanxi	460,066 (65.6)	460,066 (65.6)	
South Shaanxi	183,441 (26.1)	183,441 (26.1)	

We reported that blood donors generally have fewer inpatient and outpatients visits than non-blood donors, based on nearly 12 million person-years of follow-up in each of the subgroups (Supplementary Table 1). Of the 1.7 million blood donors, 418,312 (24.5%) and 332,569 (19.5%) individuals were outpatients and inpatients, accounting for 1,640,483 (96.2%) hospital outpatient visits and 496,061 (29.1%) inpatient visits, respectively. In contrast, of the same number of non-blood donors, a total of 407,798 (23.9%) individuals were hospital outpatients, and 346,097 (20.3%) individuals were inpatient, accounting for 1,655,725 (97.1%) hospital outpatient visits and 562,337 (33.0%) inpatient visits. The number of outpatient and inpatient visits by non-blood donors was 0.9 and 3.9% higher than those by blood donors.

**FIGURE 3:**
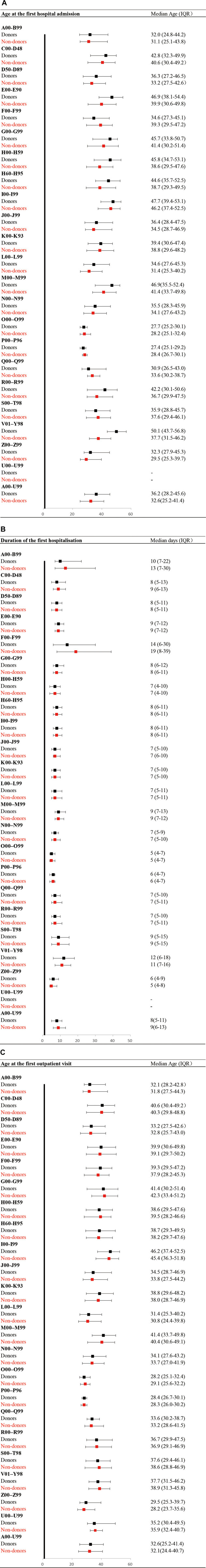
Age at first inpatient and outpatient visits and duration of the first hospitalization based on ICD-10 Chapters*, stratified by blood donors and non-blood donors. **(A)** Age at the first hospital admission. *ICD Chapters are explained in Supplementary Table 1. **(B)** Duration of the first hospitalization. **(C)** Age at the first outpatient visit.

The first inpatient and outpatient records are important indicators for the disease onset of the cohort participants. Overall, compared non-blood donors, blood donors consistently had an older age at the first inpatient visit (36.2 [28.2–45.6] vs. 35.3 [26.7–44.3], *p* < 0.001, Figure 3A and Supplementary Table 1) and at outpatient visit (32.6 [25.2–41.4] vs. 32.1 [24.4–40.7], *p* = 0.005) (Figure 3C and Supplementary Table 1), a shorter duration of the first inpatient hospitalization (8 days [5–11] vs. 9 [6–13], *p* < 0.001) (Figure 3B and Supplementary Table 1) and incurred lower costs during the first inpatient and outpatient visits (RMB ¥4684 [2,826–7,781] vs. ¥4906 [3,153–8,186], *p* < 0.001; and ¥111 [53–245] vs. ¥103 [54–237], *p* = 0.0016).

Among the blood donors, the most common cause of their first inpatient hospital visit was pregnancy, childbirth and the puerperium (26.8% of all visits, 107,263 individuals with 131,934 visits, Supplementary Table 1 and Supplementary Figure 1A), followed by diseases of the circulatory system (11.2%, 40,021 individuals with 55,229 visits), injury, poisoning and other external causes (9.8%, 43,376 individuals with 48,147 visits), digestive system diseases (9.2%, 38,951 with 45,573 visits) and diseases of the respiratory system (6.3%, 26,909 individuals with 31,215 visits). For non-blood donors, the top five disease categories are identical with only slight variation in their proportions and ranking (24.9, 11.6, 9.7, 9.8, and 6.7% for the five disease categories, respectively). In this cohort, blood donors had consistently fewer visits for all five leading diseases than non-blood donors (all *p* < 0.05, Supplementary Table 1).

The records for the first outpatient visit among blood donors demonstrated a similar pattern of distribution (Supplementary Figure 1B). The top five causes for outpatient visits were diseases of the respiratory system (35.0%, 148,889 individuals with 573,904 visits), disease of the digestive system (8.6%, 37,530 individuals with 140,642 visits), disease of the circulatory system (6.7%, 25,355 individuals with 110,021 visits), diseases of the genitourinary system (4.6%, 20,579 individuals with 76,074 visits), and diseases of the musculoskeletal system and connective tissue (4.3%, 18,449 individuals with 71,260 visits). For non-blood donors, the top five disease categories are identical with only slight variation in their proportions and ranks (38.8, 9.6, 6.9, 4.9, and 5.6% for the five disease categories, respectively). Blood donors had consistently fewer visits for the disease of the respiratory system, digestive system and musculoskeletal system and connective tissues than non-blood donors (all *p* < 0.05, Supplementary Table 1), but not diseases of the genitourinary system and the circulatory system.

We plan to further evaluate the “healthy donor effects” in a subset of participants. We will first focus on the subset of blood donors who donated blood between the 2012 baseline and the end of 2018. We will then specifically extract the disease information of these blood donors between baseline and the date of their blood donation. Similarly, we will also extract the disease information of the matching non-blood donors during the same time period. We, hence, compare the disease rates between blood donors and non-blood donors to quantify the “healthy donor effects.”

## Discussion

There are several key unique strengths of the Shaanxi Blood Donor Cohort. First, it is the largest known population cohort study for blood donors worldwide. It includes extensive details on the natural disease burdens of a population cohort in a resource-limited country undergoing rapid economic and social changes. The cohort is representative of the general population in western China, and the disease burden resembles the natural disease burden in the Chinese population (29, 30). Second, as the blood donation history is dated back to as early as 1998, this enables us to define regular and non-regular blood donors among all blood donors based on the frequency and interval of donation. Together with data from non-blood donors, this cohort provides a detailed spectrum from no blood donation to various levels of blood donation, enabling us to link and investigate the disease burden in each of these subgroups. The extensive history of blood donation records also means a large subgroup of the early blood donors has already entered the post-donation age (age > 55) during 2012–2018, providing a unique opportunity to assess blood donation’s long-term impact on donors’ health as they age. Third, the disease diagnoses are well-defined in this cohort, based on ICD-10 standards rather than self-report. It consists of all 22 categories of ICD codes from both inpatient and outpatient hospital records. The availability of detailed information on the demographics, socioeconomic status, lifestyle and dietary, medical and family disease history, blood and blood donation information at baseline allows a comprehensive assessment of the risk factors and causes of diseases. Fourth, the cohort includes a comparable subgroup of non-blood donors with identical key demographic characteristics, allowing a direct comparison of disease burden and progression between blood donors and non-blood donors. Fifth, our cohort data will enable us to quantify “healthy donor effects.” Previous studies have reported that healthy donor effects cause significant biases when assessing the association between blood donation and disease risk, but the adjustment for the effects was mostly made only after the event of blood donation (8, 31). In this study, we have designed a sub-study to explore the “healthy donor effects” before blood donation. The causative association between blood donation and subsequent disease onset can be more appropriately addressed in future analysis.

The cohort study has several limitations. First, the EHR system was only established in 2012, and the relatively short duration may limit the investigation of the onset of some chronic diseases. However, the cohort’s large sample size may compensate for this limitation by increasing the available number of person-years of follow-up. Second, the pooled data does not consider the mobility of the population; individuals who seek healthcare outside of the Shaanxi province were not reflected in the database. In 2018, the proportion of Shaanxi residents who live or work in other Chinese provinces was approximately 15% (32); this population represents a younger population and is at a lower risk of diseases. Nevertheless, the hospital diagnosis may be an underestimate of the actual number of diagnoses. Third, our study did not include menstruation nor the menopausal status of female donors as the Chinese guideline does not allow women 3 days before or after menstruation, during pregnancy, up to 6 months after abortion or childbirth and up to 1 year after lactation to donate blood (25). However, we did include menopausal women in our study, and this may be a critical factor for iron levels and lead to potential overestimation of health status in blood donors. Fourth, the hospital diagnosis depends on the medical staff’s ability to diagnose and the willingness of the individuals to seek healthcare. Mental diseases and degenerative diseases are underrepresented in this database, which accounts for only 1.0 and 0.1% of all diagnoses. Previous studies have reported a low overall level of accurate diagnosis of mental illness in the Chinese population. The misunderstanding that mental diseases are a part of aging and untreatable and the concealment of symptoms to avoid stigma from the society are main reasons for underestimating the prevalence of mental disorders (33, 34). We anticipated the situation would improve over time, and our 3-yearly ongoing follow-up will provide an opportunity to investigate these diseases in the near future. Fifth, although we have matched key demographic characteristics of the blood donor group to create the non-blood donor group, there are variations in other potential confounding factors between the two population groups. These may include socioeconomic status, family history of diseases and dietary patterns, which may contribute to the potential differences between the diseases profiles of the two groups.

## Ethics Statement

Ethical approval for the study was obtained from the institutional review board of People’s Hospital of Shaanxi Province (No: 2020-R002). The waiver of participants’ consent was obtained given that all the participants in the cohort are de-identified, and the study design is totally observed. We have already signed the agreement of waiving consent from Shaanxi Provincial People’s Hospital, Shaanxi Provincial Blood Center and Shaanxi Health Information Center according to the 25th clause from the Declaration of Helsinki. The study was registered at the Chinese Clinical Trial Registry (http://www.chictr.org.cn/index.aspx) with registration number ChiCTR2200055983. Written informed consent for participation was not required for this study in accordance with the national legislation and the institutional requirements.

## Author Contributions

LZ and HL involved in study concept and design, data acquisition, analysis, and interpretation of data and drafting of the manuscript. SS involved in the acquisition of data and interpretation of data. EW and TM involved in the interpretation of data and critical revision of the manuscript. YSu, LG, QC, XG, WW, LW, MD, and LlZ involved in the critical revision of the manuscript. YSh and JY involved in study concept and design, interpretation of data, critical revision of the manuscript, and overall study supervision. All authors participated in preparing the manuscript and have seen and approved the final version.

## Conflict of Interest

The authors declare that the research was conducted in the absence of any commercial or financial relationships that could be construed as a potential conflict of interest.

## Publisher’s Note

All claims expressed in this article are solely those of the authors and do not necessarily represent those of their affiliated organizations, or those of the publisher, the editors and the reviewers. Any product that may be evaluated in this article, or claim that may be made by its manufacturer, is not guaranteed or endorsed by the publisher.
