# Supplementary material for: Cohort Profile: The Shaanxi Blood Donor Cohort in China
Source: Front Cardiovasc Med. 2022 May 11;9:841253. doi: 10.3389/fcvm.2022.841253 (PMC9130717; doi:10.3389/fcvm.2022.841253)
Supplement: Supplementary file 1 [file Data_Sheet_1.pdf]

Supplementary Materials

Figure S1a Composition of inpatient visits among blood donors and non-blood donors

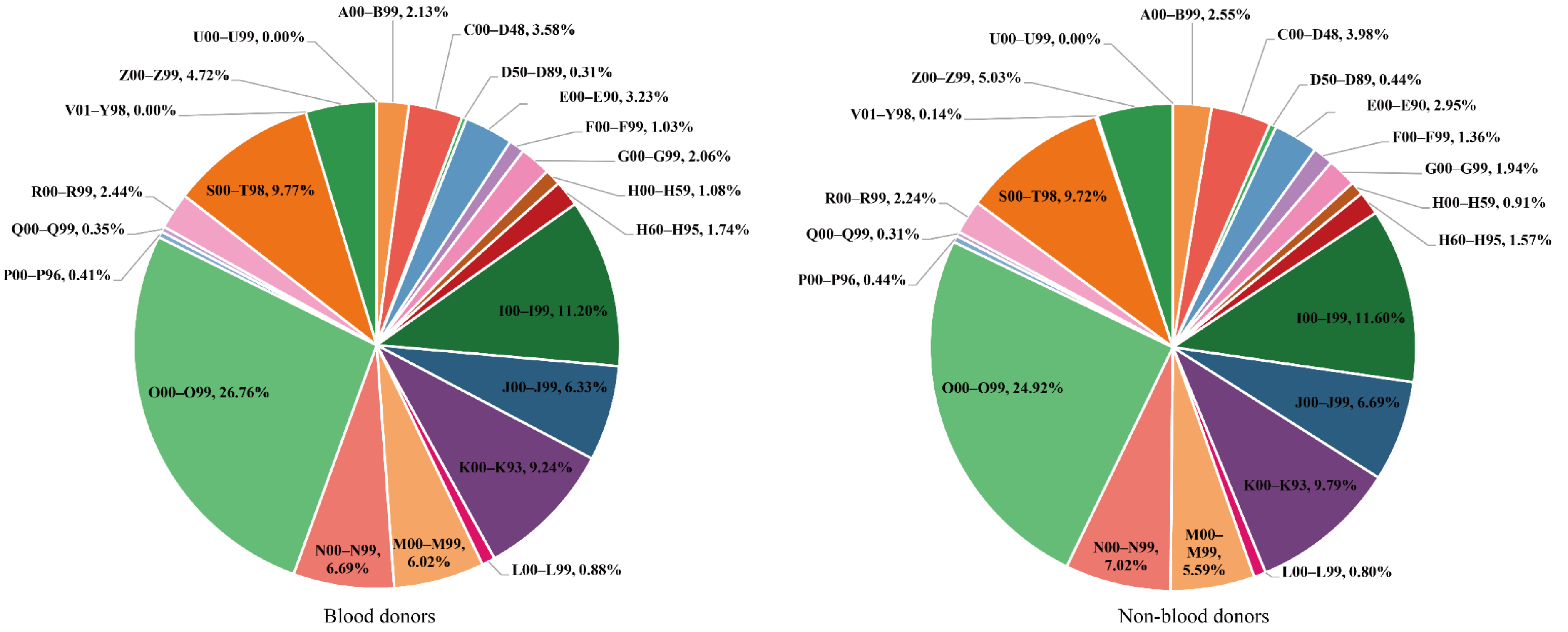

Figure S1b Composition of outpatient visits among blood donors and non-blood donors

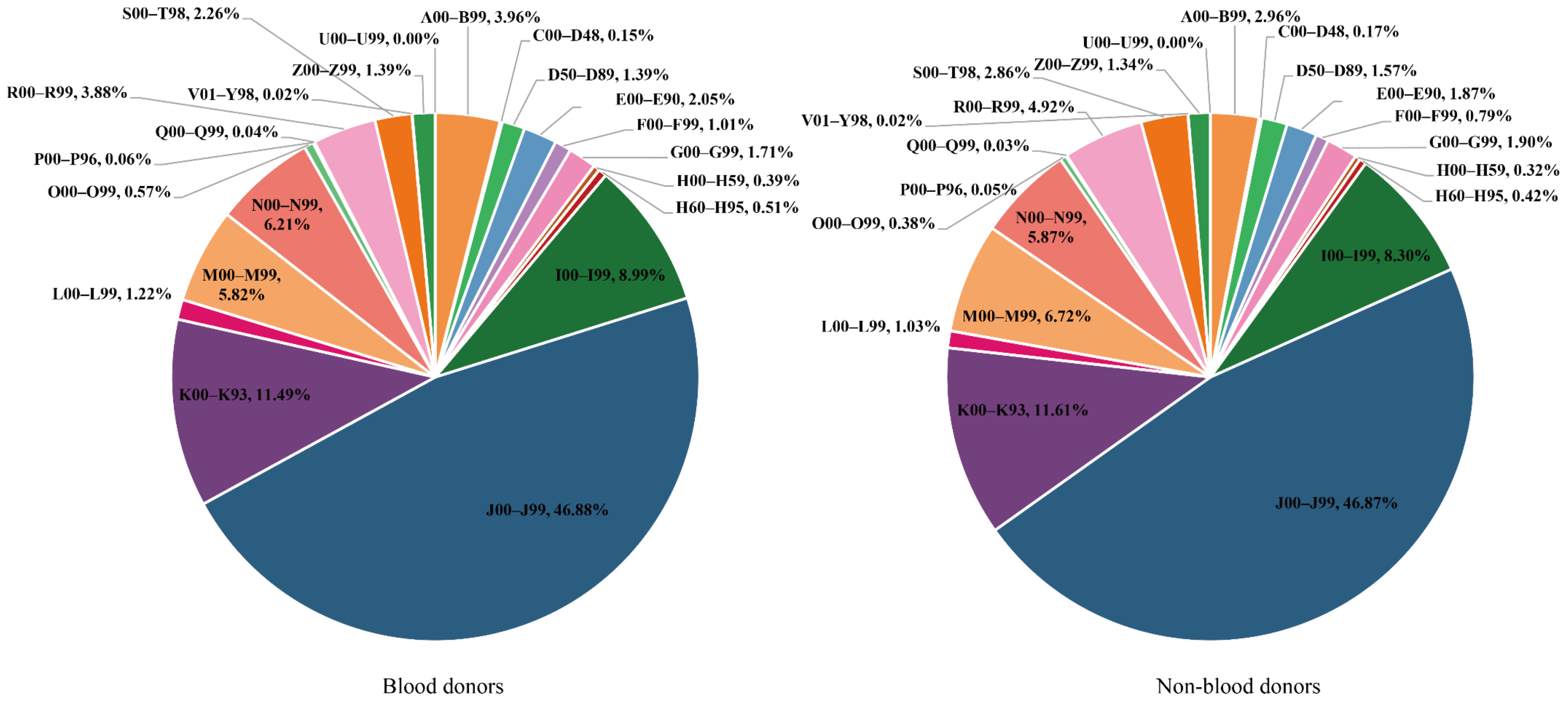

Table S1 Disease diagnosis at first inpatient and outpatient visits based on ICD-10 classification, stratified by blood donors and non-blood donors

		Blood donors					Non-blood donors				
Chapter/Block	Category	Inpatients (persons/visits)	Age at the first hospital admission	Duration of the first hospitalisation	Outpatients (persons/visits)	Age at the first outpatient visit	Inpatients (persons/visits)	Age at the first hospital admission	Duration of the first hospitalisation	Outpatients (persons/visits)	Age at the first outpatient visit
I: A00–B99	Certain infectious and parasitic diseases	8758/10509	32.0 (24.8-44.2)	10 (7-22)	29004/48437	32.1 (28.2-42.8）	10461/13390	31.1 (25.1-43.8)	13 (7-30)	21892/40500	31.8 (27.5-44.3)
II: C00–D48	Neoplasms	12798/17661	42.8 (32.3-49.9)	8 (5-13)	1335/1776	40.6 (30.4-49.2）	14298/20875	42.1 (32.0-49.6)	9 (6-13)	1080/2323	40.3 (29.8-48.8)
III: D50–D89	Diseases of the blood and blood-forming organs and certain disorders involving the immune mechanism	1266/1545	36.3 (27.2-46.5)	8 (5-11)	11906/17026	33.2 (27.5-42.6）	1835/2330	36.1 (27.0-46.9)	8 (5-11)	13866/21493	32.8 (25.7-43.0)
IV: E00–E90	Endocrine, nutritional and metabolic diseases	11718/15937	46.9 (38.1-54.4)	9 (7-12)	14658/25066	39.9 (30.6-49.8)	10659/15456	46.4 (38.7-53.9)	9 (7-12)	14614/25575	39.1 (29.7-50.2)
V: F00–F99	Mental and behavioural disorders	3373/5093	34.6 (27.3-45.1)	14 (6-30)	8561/12414	39.3 (29.5-47.2)	3780/7107	34.3 (27.8-44.9)	19 (8-39)	6484/10831	37.9 (28.2-45.3)
VI: G00–G99	Diseases of the nervous system	8827/10151	45.7 (33.8-50.7)	8 (6-12)	14164/20963	41.4 (30.2-51.4)	8478/10173	46.1 (34.5-52.3)	8 (6-11)	17819/26060	42.3 (33.4-51.2)
VII: H00–H59	Diseases of the eye and adnexa	4429/5315	45.8 (34.7-53.1)	7 (4-10)	3948/4817	38.6 (29.5-47.6)	3894/4751	45.9 (34.4-53.6)	7 (4-10)	3496/4416	39.5 (28.2-46.6)
VIII: H60–H95	Diseases of the ear and mastoid process	7671/8592	44.6 (35.7-52.5)	8 (6-11)	4337/6245	38.7 (29.3-49.5)	7160/8234	44.0 (35.1-53.2)	8 (6-11)	4087/5768	38.2 (29.7-47.6)
IX: I00–I99	Diseases of the circulatory system	40021/55229	47.7 (39.4-53.1)	8 (6-11)	50238/110021	46.2 (37.4-52.5)	39502/60833	47.4 (39.2-54.6)	8 (6-11)	51857/113599	45.4 (36.3-51.8)
X: J00–J99	Diseases of the respiratory system	26909/31215	36.4 (28.4-47.5)	7 (5-10)	206440/573904	34.5 (28.7-46.9)	29226/35071	35.0 (27.8-45.4)	7 (6-10)	223238/641813	33.8 (27.5-44.2)
XI: K00–K93	Diseases of the digestive system	38951/45573	39.4 (30.6-47.4)	7 (5-10)	81769/140642	38.8 (29.6-48.2)	40112/51343	39.1 (30.6-47.0)	7 (5-10)	87618/159007	38.0 (28.7-46.9)
XII: L00–L99	Diseases of the skin and subcutaneous tissue	3797/4329	34.6 (27.6-45.3)	7 (5-11)	10880/14905	31.4 (25.3-40.2)	3583/4192	33.1 (26.4-44.2)	7 (5-11)	10163/14125	30.8 (24.4-39.8)
XIII: M00–M99	Diseases of the musculoskeletal system and connective tissue	24125/29674	46.9(35.5-52.4)	9 (7-13)	44261/71260	41.4 (33.7-49.8)	22922/29340	46.2 (35.3-51.8)	9 (7-12)	52955/92052	40.4 (30.6-49.1)
XIV: N00–N99	Diseases of the genitourinary system	26613/33000	35.5 (28.3-45.9)	7 (5-9)	40465/76074	34.1 (27.6-43.2)	27081/36830	34.5 (27.8-45.3)	7 (5-10)	40998/80425	33.7 (27.0-41.9)
XV: O00–O99	Pregnancy, childbirth and the puerperium	107263/131934	27.7 (25.2-30.1)	5 (4-7)	4920/7036	28.2 (25.1-32.4)	104568/130710	27.0 (24.5-29.8)	5 (4-7)	3396/5151	29.1 (25.6-32.2)
XVI: P00–P96	Certain conditions originating in the perinatal period	1946/2024	27.4 (25.1-29.2)	6 (4-7)	565/695	28.4 (26.7-30.1)	2163/2293	27.8 (24.3-29.4)	6 (4-7)	521/641	28.3 (26.0-30.2)
XVII: Q00–Q99	Congenital malformations, deformations and chromosomal abnormalities	1588/1731	30.9 (26.5-43.0)	7 (5-10)	460/538	33.6 (30.2-38.7)	1455/1615	30.5 (25.9-42.6)	7 (5-11)	292/354	33.2 (28.6-41.5)
XVIII: R00–R99	Symptoms, signs and abnormal clinical and laboratory findings, not elsewhere classified	10628/12010	42.2 (30.1-50.6)	7 (5-10)	32322/47514	36.7 (29.9-47.5)	10147/11771	42.0 (29.7-50.1)	7 (5-11)	41556/67404	36.9 (29.1-46.9)
XIX: S00–T98	Injury, poisoning and certain other consequences of external causes	43376/48147	35.9 (28.8-45.7)	9 (5-15)	20468/27632	37.6 (29.4-46.1)	44310/50957	37.1 (29.8-45.4)	9 (5-15)	26969/39208	38.6 (28.8-46.9)
XX: V01–Y98	External causes of morbidity and mortality	3/3	50.1 (43.7-56.8)	12 (6-18)	228/297	37.7 (31.5-46.2)	542/754	51.1 (41.8-60.5)	11 (7-16)	174/217	38.9 (31.3-45.8)
XXI: Z00–Z99	Factors influencing health status and contact with health services	12452/23286	32.3 (27.9-45.3)	6 (4-9)	11086/16962	29.5 (25.3-39.7)	15900/26394	30.5 (26.4-39.8)	5 (4-8)	11341/18380	28.2 (23.7-35.6)
XXII: U00–U99	Codes for special purposes	0/0	-	-	5/11	35.2 (30.4-49.5)	0/0	-	-	21/52	35.9 (32.4-40.7)
**A00-U99**	**All diseases**	332569/496061	36.2 (28.2-45.6)	8(5-11)	418312/1640483	32.6(25.2-41.4)	346097/562337	35.3(26.7-44.3)	9(6-13)	407798/1655725	32.1(24.4-40.7)

Table S2: Centralised hospital medical record coverage rate in specialised hospitals and primary hospitals.

Centralised hospital medical record coverage rate		
Year \ Hospital	Specialised hospital	Primary hospital
2012	80.0%	30.0%
2013	90.5%	40.5%
2014	93.0%	50.3%
2015	96.0%	62.4%
2016	98.5%	70.0%
2017	100.0%	74.6%
2018	100.0%	80.0%
